# Relationship of Biochemical and Sonographic Markers with Disease Severity in Rosacea Patients Without Cardiovascular Disease

**DOI:** 10.3390/life15010046

**Published:** 2025-01-01

**Authors:** Banu Ismail Mendi, Bokebatur Ahmet Rasit Mendi, Banu Farabi, Mehmet Fatih Atak

**Affiliations:** 1 Department of Dermatology, Nigde Omer Halisdemir University, Nigde 51000, Turkey; banu.mendi@saglik.gov.tr; 2 Department of Radiology, Nigde Omer Halisdemir University, Nigde 51000, Turkey; b.mendi@saglik.gov.tr; 3 Department of Dermatology, New York Medical College, New York, NY 10595, USA; fatih9164@hotmail.com; 4 Dermatology Department, NYC Health + Hospital/Metropolitan, New York, NY 10029, USA; 5 Dermatology Department, NYC Health + Hospital/South Brooklyn, New York, NY 11235, USA

**Keywords:** rosacea, atherosclerosis, cardiovascular diseases, visceral adipose tissue, sedimentation

## Abstract

Rosacea is a chronic inflammatory skin disorder characterized by central facial redness, papulopustular lesions, and occasionally phymatous changes. There is ongoing debate regarding rosacea as a cutaneous disease with systemic inflammatory effects and its associations with cardiovascular diseases. Although the pathogenesis of both atherosclerosis and rosacea demonstrate notable similarities, particularly in the central role of inflammation, significant gaps in understanding these connections remain. This study aims to investigate this potential relationship by assessing biochemical markers and sonographic findings in rosacea patients who were divided into groups based on disease severity. Our study included 73 rosacea patients and 73 age- and sex-matched controls, without cardiovascular disease. Demographic, clinical, and laboratory data were recorded for all participants. Carotid intima–media thickness and the thickness of subcutaneous, preperitoneal, posterior perirenal, and aortic-level visceral adipose tissues were measured by ultrasonography. The erythrocyte sedimentation rate was significantly elevated in rosacea patients versus controls. Additionally, sonographic assessments revealed that both aortic-level visceral adipose tissue and preperitoneal adipose tissue were significantly thicker in rosacea patients than in the control group, a finding corroborated by multivariable linear regression analysis. While thicker preperitoneal and perirenal adipose tissues were observed with increasing disease severity, these differences did not achieve statistical significance when subjected to multivariable linear regression analysis. The thorough examination and follow-up of patients with rosacea for cardiovascular risk factors may be necessary in clinical practice.

## 1. Introduction

Rosacea is a chronic inflammatory skin condition characterized by episodic flushing followed by persistent central erythema, telangiectasias, red papules, and pustules, and in some cases, phymatous or granulomatous changes. Ocular symptoms can also be associated with the condition. Rosacea predominantly affects fair-skinned women over the age of thirty [1]. Although numerous studies have been conducted on the etiopathogenesis of rosacea, genetic predisposition, neurovascular dysregulation, activation of the innate immune system, and environmental factors are believed to play significant roles [2].

There are perspectives suggesting that rosacea has systemic inflammatory implications rather than just being a skin disorder, and it may be associated with various systemic diseases [2]. Numerous studies have been conducted to assess the comorbidities associated with rosacea. Research indicates that rosacea is linked to various conditions, including thyroid disorders, cardiovascular diseases, gastrointestinal disorders, neurological conditions, and malignancies [3,4].

The relationship between rosacea and cardiovascular diseases remains controversial [5,6]. Research has identified shared pathogenic mechanisms between rosacea and atherosclerosis, such as increased oxidative stress, higher levels of cathelicidin in inflammatory cells, and lower levels of anti-inflammatory agents [2,3]. Furthermore, chronic systemic inflammation is believed to trigger and exacerbate the atherosclerotic process through endothelial dysfunction [7]. Evidence from the literature indicates that chronic inflammatory conditions, including rheumatoid arthritis, psoriasis, and rosacea, are associated with an elevated risk of cardiovascular diseases [8,9]. One study demonstrated that rosacea treatment with oral tetracycline decreased the occurrence of vascular events, possibly due to the drug’s anti-inflammatory properties [10]. Additionally, neurovascular dysregulation, a key factor in the pathogenesis of rosacea, is implicated in the development of hypertension, a form of cardiovascular disease [11].

Increased visceral adipose tissue (VAT) contributes to the progression of atherosclerosis independently of obesity by promoting the release of proinflammatory cytokines [12,13]. Carotid intima–media thickness (CIMT) is associated with subclinical atherosclerosis and serves as an important marker in assessing cardiovascular risk [14].

In this study, we aimed to evaluate the relationship between rosacea and biochemical parameters, VAT, and CIMT values.

## 2. Materials and Methods

### 2.1. Study Population

We conducted a cross-sectional study involving rosacea patients aged 18 years and older, as well as age- and gender-matched controls, at the Department of Dermatology, Nigde Omer Halisdemir University Training and Research Hospital, between February 2024 and August 2024. Prior to the study, we obtained approval from the ethics committee. Participants in both the rosacea and control groups were selected consecutively based on specific inclusion and exclusion criteria.

Patients and controls meeting any of the following criteria were excluded from the study: those with a history of cardiac and/or cerebrovascular disease, diabetes mellitus (defined as a fasting blood sugar level ≥126 mg/dL), hypertension (systolic blood pressure ≥ 140 mmHg and/or diastolic blood pressure ≥ 90 mmHg), dyslipidemia (total cholesterol level ≥ 200 mg/dL, triglyceride level ≥ 150 mg/dL, or high-density lipoprotein [HDL] cholesterol level ≤ 40 mg/dL), or chronic kidney disease (glomerular filtration rate < 60); those with a diagnosis of inflammatory dermatological and/or connective tissue and/or inflammatory bowel disease; obese individuals (body mass index > 30); those with a history of smoking; those using medications affecting the cardiovascular system (such as antihypertensive, antihyperlipidemic, antidiabetic, or anticoagulant drugs); and those with only ocular involvement. Rosacea diagnosis and subtype classifications were made clinically according to the criteria and guidelines established by the National Rosacea Society Expert Committee [15]. Therefore, steroid-induced acneiform eruptions, periorificial dermatitis, and pure demodicosis cases without inflammation were excluded from the study.

Rosacea patients were divided into 3 subtypes: erythematotelangiectatic rosacea (ETR), papulopustular rosacea (PPR), and phymatous rosacea (PR). Disease severity was assessed according to the rosacea clinical severity index proposed by the National Rosacea Society Expert Committee [15]. Aspartate aminotransferase (AST), alanine aminotransferase (ALT), sedimentation rate (ESR), C-reactive protein (CRP), and complete blood count values of patients and controls were recorded.

### 2.2. Imaging Procedures

**Figure 1:**
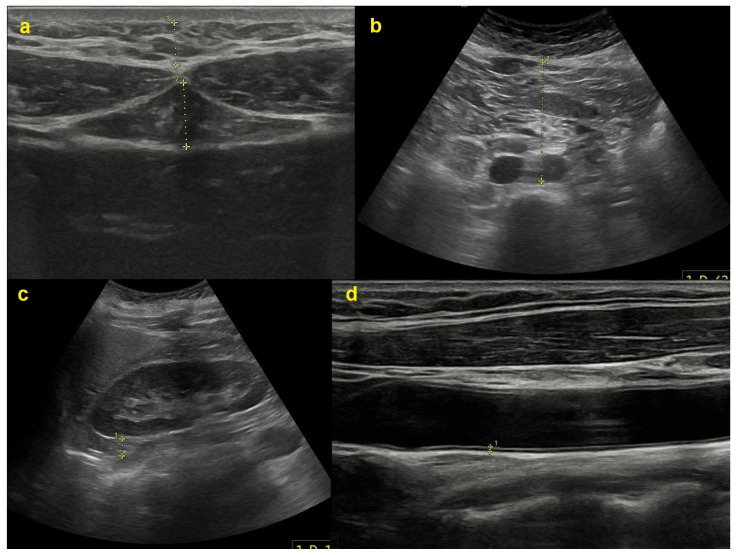
Sonographic measurements of various adipose tissue thicknesses as well as the intima–media thickness of a carotid artery. The (**a**) subunit reflects the subcutaneous (**upper**) and preperitoneal (**lower**) fat tissue thickness at the xiphoid process level; (**b**) the distance between the aorta and the anterior abdominal wall; (**c**) the posterior perirenal fat tissue thickness; and (**d**) the carotid intima–media thickness.

Sonographic measures were acquired by a radiologist utilizing an ultrasound device (Samsung Medison V8; Samsung Healthcare, Seoul, Republic of Korea). A 3.5 MHz curved-array probe was employed to assess adipose tissue parameters ’a’, ’b’, ’c’, and ’d’ (Figure 1), as detailed below: a—thickness of subcutaneous adipose tissue at the xiphoid level; b—thickness of preperitoneal adipose tissue at the xiphoid level; c—distance between the posterior wall of the aorta and the internal surface of the rectus abdominis muscle at the level of the umbilicus (aorta VAT); and d—thickness of right posterior perirenal adipose tissue (perirenal VAT) [16]. Parameters ’c’ and ’d’ pertain to VAT.

Furthermore, CIMT was assessed utilizing an 11 MHz linear-array probe on the same device. CIMT values were assessed at the conclusion of diastole and analyzed in the longitudinal view, focusing on non-plaque segments of each common carotid artery. All measurements were acquired after an overnight fast, essential for abdominal parameter assessment, and all data were collected in the supine position at the end of expiration. To avoid compressing adipose tissue, a curvilinear transducer was placed perpendicular to the skin with minimal pressure.

### 2.3. Statistical Analyses

Statistical analyses were conducted using Jamovi Statistics Software (Version 2.3.28.0). Descriptive statistics for the study population are reported as proportions, means, or medians, depending on the data distribution. The normality of numeric variables was assessed using the Shapiro–Wilk test and Q-Q plots. Comparisons of quantitative variables were performed using either Student’s t-test or the Mann–Whitney U test, based on the distribution of the data. Qualitative variables were analyzed using the Pearson chi-square test or Fisher’s exact test, as appropriate. Multivariable linear regression models were employed to assess the association of numerical or categorical independent variables with continuous dependent variables. A significance threshold of 0.05 was applied for all statistical tests.

## 3. Results

**Figure 2:**
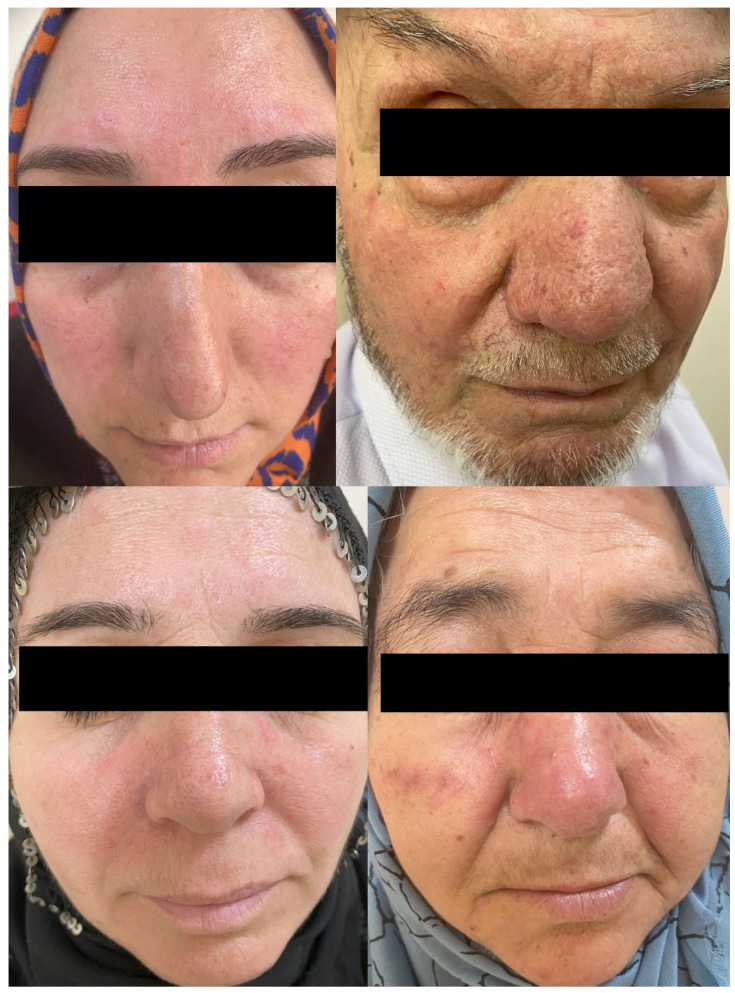
Illustrative facial images of the group of rosacea patients.

A total of 73 rosacea patients and 73 age- and sex-matched controls were included in the study. Demographic, clinical, and laboratory data for the patients and controls are presented in Table 1. There was no significant difference in BMI between the control and patient groups but the patient group had significantly lighter skin types according to the Fitzpatrick scale. Fifty-one (69.9%) of the patients had ETR, 20 (27.4%) had PPR, and 2 (2.7%) had PR. According to the disease severity index (9), 19 (26.0%) of the patients had mild disease, 48 (65.8%) had moderate disease, and 6 (8.2%) had severe disease. Seventeen (23.3%) of the patients also had ocular involvement. Sample facial images from the rosacea patient group included in the study are shown in Figure 2.

The mean ESR value in rosacea patients was significantly higher than in the control group (15.10 ± 10.8 vs. 6.86 ± 3.05; *p* < 0.01). No significant difference was found in terms of CRP, lymphocyte, neutrophil, and platelet counts, mean platelet volume, neutrophil/lymphocyte ratio (NLR), and platelet/lymphocyte ratio.

According to sonographic findings, preperitoneal adipose tissue at the xiphoid process level (14.11 ± 4.47 vs. 11.0 ± 3.12; *p* < 0.001) and aorta VAT (36.9 ± 16.26 vs. 25.57 ± 6.32; *p* < 0.001) were found to be significantly thicker in rosacea patients compared to the control group. However, no significant differences existed in CIMT, subcutaneous adipose tissue, and perirenal VAT thickness.

We evaluated demographic, laboratory, and sonographic findings based on disease severity, subtype, and ocular involvement (Table 2). Patients were categorized into mild and moderate–severe groups according to disease severity. In the moderate–severe group, age (39.4 ± 11.5 vs. 32.8 ± 9.4; *p* = 0.02) and BMI (25.3 ± 2.5 vs. 23.8 ± 2.8; *p* = 0.03) were significantly higher, and preperitoneal adipose tissue (14.9 ± 4.2 vs. 11.6 ± 4.2; *p* = 0.008) and perirenal VAT (8.5 ± 1.9 vs. 7.4 ± 1.4 *p* = 0.049) were notably thicker compared to in the cohort with mild disease. The findings did not reveal significant differences concerning ocular involvement and disease subtype.

When the significant sonographic findings were re-evaluated using multivariable linear regression analysis, aorta VAT and preperitoneal fat tissue at the xiphoid level remained significant (*p* < 0.001). However, the association between rosacea severity and both preperitoneal adipose tissue and perirenal VAT was not significant. The results of multivariable linear regression analysis are shown in Table 3. The parameters found to be significant in the study are summarized in Table 4.

## 4. Discussion

Rosacea is predominantly a cutaneous disease, yet it also exhibits systemic inflammatory markers, some of which are linked to cardiovascular diseases [2,4]. It involves the activation of the innate and adaptive immune systems, including cells such as keratinocytes, macrophages, neutrophils, mast cells, helper T cells, fibroblasts, and vascular endothelium cells. These cells release molecules such as IL-1β, tumor necrosis factor, matrix metalloproteinases, reactive oxygen species, and the antimicrobial peptide cathelicidin, all contributing to inflammation in rosacea [2]. Demodex, an additional factor in the pathogenesis of rosacea, triggers inflammation by damaging the follicular epithelium through the lipases and proteases it secretes and by depleting the exoskeletal material along with post-mortem internal components [17]. Beyond the inflammation initiated by Demodex mites themselves, it has been established that the symbiotic bacteria they harbor, Bacillus oleronius, Bacillus pumilus, and Bacillus cereus, further exacerbate inflammatory responses [18,19,20]. Clinical investigations have consistently identified higher levels of inflammatory markers in rosacea patients compared to control groups. For instance, Karaosmanoglu et al. found significantly elevated levels of ESR, CRP, MPV, and SII index values in the blood of rosacea patients [21]. Similarly, a study by Ertekin et al. reported elevated levels of serum proinflammatory cytokines, including high-sensitivity CRP (hs-CRP), TNF-α, IL-1β, and IL-6, compared to controls [22]. Our study corroborates these findings, showing that ESR values are significantly higher in patients with rosacea than in the control group.

Following the skin, the eyes are the second most commonly affected organ in rosacea. Research indicates elevated levels of proinflammatory cytokines such as IL-1a, along with reduced levels of anti-inflammatory cytokines such as IL-10 in the tears of rosacea patients with ocular symptoms [23,24]. Furthermore, Demodex mites, which colonize the meibomian glands of the eye, appear to be a potent source of inflammation; when deeply embedded, their chitinous exoskeleton can provoke a granulomatous foreign body response [17]. According to the literature, the prevalence of ocular rosacea exhibits considerable variability, with rates ranging from 6% to 58% [25,26,27,28]. In one study from Turkey, researchers found a 23.3% incidence of ocular involvement in patients with cutaneous rosacea after excluding those with isolated ocular rosacea [29]. Our study, applying similar exclusion criteria, also identified an ocular involvement rate of 23.3% among patients exhibiting dermatological symptoms of rosacea, aligning with results from within this geographic region. Variations among the studies could be attributable to racial and geographic differences, as well as to the absence of definitive diagnostic criteria for ocular rosacea [30].

Inflammation is an important factor at every stage of atherosclerosis and its complications. During the early phases of atherosclerosis, monocytes that have ingested oxidized lipids migrate to the affected area and differentiate into macrophages and foam cells, thereby forming fatty streaks. Subsequently, the influx of additional inflammatory mediators facilitates the formation of an atheroma plaque. This plaque becomes prone to rupture as collagen is degraded by metalloproteinases secreted by monocytes and macrophages. Recent research has identified that similar biological pathways are involved in both rosacea and atherosclerosis [31]. Notably, cathelicidin increases, and the activity of the antioxidant enzyme serum paraoxonase-1 (PON), which metabolizes lipid peroxides and prevents oxidative modification of serum lipoproteins, decreases during both conditions [3]. Cathelicidin levels, known for their proinflammatory attributes, leading to an enhanced release of cytokines and chemokines, have been found to be elevated in rosacea patients. This peptide plays a dual role in antimicrobial defense and in stimulating the innate immune system through various pathways, thereby promoting endothelial cell proliferation and angiogenesis [32]. Additionally, null mutations in the GSTM1 and GSTT1 gene loci, which encode glutathione-S-transferase—an enzyme that catalyzes toxic oxidative intermediates—were more frequently observed in rosacea patients compared to the control group [2]. Given these findings, cardiovascular risk factors have been explored in rosacea patients. A 2014 case–control study revealed that individuals with rosacea had higher incidences of dyslipidemia, smoking, alcohol consumption, and a family history of cardiovascular disease compared to control subjects [33]. Similarly, a case–control study conducted in Taiwan identified associations between rosacea and conditions such as dyslipidemia, coronary artery disease, and hypertension. The link between coronary artery disease and rosacea remained significant even after adjusting for diabetes, hypertension, and dyslipidemia [5]. In contrast, a study by Egeberg et al. reported no significant association between rosacea and cardiovascular risk factors or mortality [6]. A 2020 meta-analysis indicated that rosacea is associated with hypertension and dyslipidemia, but not with ischemic heart disease, stroke, or diabetes [11]. More recently, a large-scale study in Korea using the national health system database followed both rosacea patients and non-rosacea controls and discovered that individuals with rosacea had significantly higher incidences of cardiovascular and coronary heart diseases post-diagnosis compared to the control group. This relationship remained significant even after accounting for risk factors like smoking, alcohol consumption, body mass index, and comorbidities [4].

The conflicting findings regarding the relationship between rosacea and cardiovascular disease have led researchers to investigate the objective measures of atherosclerosis, which underlies cardiovascular conditions [34]. Belli et al. examined epicardial fat tissue and CIMT as indicators of subclinical atherosclerosis in rosacea patients and discovered that both measures were significantly elevated compared to the control group [35]. Conversely, in another study, Ertekin et al. assessed the CIMT in both rosacea patients and control groups but found no significant differences between them [22]. Additionally, Caf et al. measured flow-mediated dilation (FMD) in rosacea patients to assess endothelial dysfunction, an early indicator of atherosclerosis. They observed that FMD, which negatively correlates with endothelial dysfunction, was significantly reduced in rosacea patients [29].

Adipose tissue functions as a secretory organ, producing adipokines and proinflammatory cytokines [36]. Although obesity is recognized as a cardiovascular risk factor, the importance of adipose tissue distribution and the association between increased visceral adipose tissue and atherosclerosis have also been emphasized in non-obese patients. Research has indicated that VAT is linked to atherosclerosis in non-obese individuals [37,38]. Additionally, preperitoneal adipose tissue has been identified as associated with atherosclerosis and is considered a critical parameter for the early prediction of this condition [38]. In our study, we examined subcutaneous adipose tissue, preperitoneal adipose tissue, CIMT, and VAT values from two areas in rosacea patients without cardiovascular risk factors. Both VAT and preperitoneal adipose tissue thickness were significantly higher in rosacea patients compared to the control group. These findings retained their significance upon confirmation through multivariable linear regression analysis. When we analyzed our parameters based on the severity of rosacea, we observed an increase in VAT and preperitoneal fat tissue thickness with increasing severity of rosacea, although this trend did not reach statistical significance in the multivariable regression analysis. Consistent with the existing literature, our findings support the association between rosacea and atherosclerosis. Nonetheless, we did not observe a significant impact of rosacea severity or subtype on this association.

Numerous research studies have assessed the correlation between ocular involvement in rosacea, associated comorbidities, and inflammatory markers. Duman et al., while finding an association between rosacea and dyslipidemia, did not identify any correlation between ocular involvement and both dyslipidemia and diabetes when evaluated independently [33]. Similarly, Caf et al., in their study investigating the link between rosacea and subclinical atherosclerosis via the FMD technique, concluded that ocular involvement exhibited no discernible impact in this context [29]. Conversely, Ertekin et al. identified a correlation between CIMT and Hs-CRP, a marker indicative of a proatherogenic state, and the presence of ocular symptoms in rosacea patients [22]. Aligning with the findings observed by Caf and Duman, we found no association between ocular involvement and inflammatory markers, or with CIMT and VAT parameters.

Our study has certain limitations. The study’s scope is limited by its relatively small sample size and its design as a single-center investigation. Secondly, owing to the recruitment process being conducted within a dermatology clinic, it precluded the inclusion of patients who solely exhibit ocular symptoms. Finally, since it is unclear whether rosacea and demodicosis-related rosacea are two separate entities [20,39,40], we did not separate these two conditions in our study.

## 5. Conclusions

In our study, we observed that ESR was elevated in comparison to the control group, reinforcing the concept that rosacea has systemic inflammatory effects. Additionally, the thicknesses of visceral and preperitoneal fat tissues, which are critical in predicting atherosclerosis, were found to be increased in rosacea patients relative to controls. While these results support the link between rosacea and cardiovascular diseases, further research with a larger cohort is warranted to substantiate this relationship. Given these findings, we recommend close monitoring for cardiovascular risk factors in this patient group.

## Figures and Tables

**Table 1:** Distribution of the obtained parameters and demographic data between the rosacea and control groups.

Variables	Rosacea, *n* = 73	Control, *n* = 73	*p* Value
Age, year, mean ± SD	37.7 ± 11.3	37.7 ± 11.3	0.99
Men/Women, *n*	9/64	9/64	1
Fitzpatrick skin type, *n* (%) I II III IV	3 (4.1%) 21 (28.8%) 35 (47.9%) 14 (19.2%)	0 (0%) 12 (16.4%) 36 (49.3%) 25 (34.2%)	0.036
Subtype of rosacea, *n* (%) ET PP Phymatous	51 (69.9%) 20 (27.4%) 2 (2.7%)		
Disease severity Mild Moderate Severe	19 (26.0%) 48 (65.8%) 6 (8.2%)		
Eye involvement, *n* (%) Yes No	17 (23.3%) 56 (76.7%)		
BMI, kg/m^2^, mean ± SD	25.0 ± 2.67	25.2 ± 2.80	0.62
ESR mm/first h, mean ± SD	15.10 ± 10.8	6.86 ± 3.05	<0.01
CRP, mg/L, mean ± SD	3.03 ± 3.16	2.97 ± 2.64	0.77
Neutrophil count, K/mL, mean ± SD	4.30 ± 1.44	4.29 ± 1.38	0.91
Lymphocyte count, K/mL, mean ± SD	2.51 ± 0.61	2.61 ± 0.64	0.35
Platelet count, K/mL, mean ± SD	323.986 ± 60.390	325.575 ± 58.896	0.87
NLR, mean ± SD	1.74 ± 0.56	1.68 ± 0.52	0.53
PLR, mean ± SD	134.19 ± 36.44	130.27 ± 35.79	0.51
MPV, mean ± SD	10.35 ± 0.88	10.38 ± 0.74	0.69
Subcutaneous fat—xiphoid, mm, mean ± SD	14.09 ± 5.09	14.09 ± 5.01	0.99
Preperitoneal fat—xiphoid, mm, mean ± SD	14.11 ± 4.47	11.0 ± 3.12	<0.001
Aorta VAT, mm, mean ± SD	36.9 ± 16.26	25.57 ± 6.32	<0.001
Perirenal VAT, mm, mean ± SD	8.23 ± 1.86	8.13 ± 1.77	0.84
Mean carotid IMT, mm, mean ± SD	0.77 ± 0.19	0.77 ± 0.21	0.94

SD: standard deviation; ET: erythematotelangiectatic subtype; PP: papulopustular subtype; BMI: body mass index; ESR: erythrocyte sedimentation rate; CRP: C-reactive protein; NLR: neutrophil-to-lymphocyte ratio; PLR: platelet-to-lymphocyte ratio; MPV: mean platelet volume; VAT: visceral adipose tissue; IMT: intima–media thickness.

**Table 2:** Distribution of obtained parameters and demographic data according to disease severity, eye involvement, and disease subtype.

	Disease Severity			Eye Involvement			Rosacea Subtype		
Variables	Mild (19)	Moderate–Severe (54)	*p* Value	Yes (17)	No (56)	*p* Value	ET (51)	PP (20)	*p* Value
Age, year, mean ± SD	32.8 ± 9.4	39.4 ± 11.5	0.02	39.9 ± 10.6	37.0 ± 11.5	0.35	37.4 ± 11.6	36.8 ± 10.0	0.88
Sex Men Women	2 (10.5%) 17 (89.5%)	7 (13%) 47 (87%)	0.78	1 (5.1%) 16 (94.1%)	8 (14.3%) 48 (85.7%)	0.35	6 (11.8%) 45 (88.2%)	3 (15%) 17 (85%)	0.71
BMI, kg/m^2^, mean ± SD	23.8 ± 2.8	25.3 ± 2.5	0.03	25.4 ± 2.1	24.8 ± 2.8	0.37	24.5 ± 2.6	25.7 ± 2.3	0.07
ESR mm/first h, mean ± SD	17.0 ± 16.5	14.4 ± 8.0	0.99	15.7 ± 6.2	14.9 ± 11.9	0.17	15.7 ± 11.7	13.1 ± 8.5	0.35
CRP, mg/L, mean ± SD	3.3 ± 2.9	2.92 ± 3.2	0.84	2.9 ± 2.1	3.0 ± 3.4	0.50	3.2 ± 3.3	2.4 ± 2.7	0.15
Neutrophil count, K/mL, mean ± SD	4.0 ± 0.9	4.4 ± 1.5	0.57	4.4 ± 1.6	4.2 ± 1.3	0.91	4.2 ± 1.4	4.6 ± 1.4	0.21
Lymphocyte count, K/mL, mean ± SD	2.4 ± 0.4	2.5 ± 0.6	0.54	2.4 ± 0.5	2.5 ± 0.6	0.51	2.4 ± 0.5	2.6 ± 0.7	0.48
Platelet count, K/mL, mean ± SD	305.8 ± 61.0	330.3 ± 59.4	0.12	344.5 ± 44.4	317.7 ± 63.4	0.10	318.9 ± 59.9	338.5 ± 61.9	0.22
NLR, mean ± SD	1.66 ± 0.47	1.77 ± 0.60	0.62	1.68 ± 0.35	1.76 ± 0.41	0.58	1.72 ± 0.54	1.78 ± 0.47	0.49
PLR, mean ± SD	127 ± 25.76	136.7 ± 39.4	0.37	124.8 ± 33.2	138.3 ± 36.5	0.33	131.4 ± 29.2	137.2 ± 38.1	0.41
MPV, mean ± SD	10.6 ± 1.20	10.28 ± 0.72	0.33	10.2 ± 0.91	10.55 ± 0.7	0.44	10.6 ± 1.1	10.15 ± 0.85	0.52
Subcutaneous fat—xiphoid, mm, mean ± SD	12.9 ± 5.5	14.5 ± 4.8	0.31	15.1 ± 6.2	13.7 ± 4.7	0.34	13.6 ± 4.9	14.5 ± 5.1	0.50
Preperitoneal fat—xiphoid, mm, mean ± SD	11.6 ± 4.2	14.9 ± 4.2	0.008	14.6 ± 5.3	13.9 ± 4.2	0.57	13.5 ± 4.2	14.8 ± 4.7	0.28
Aorta VAT, mm, mean ± SD	31.7 ± 10.4	38.7 ± 17.6	0.22	37.4 ± 16.7	36.7 ± 16.2	0.88	34.6 ± 16.1	39.9 ± 14.8	0.12
Perirenal VAT, mm, mean ± SD	7.4 ± 1.4	8.5 ± 1.9	0.049	8.8 ± 2.1	8.0 ± 1.7	0.24	8.0 ± 1.7	8.3 ± 1.8	0.35
Carotid, mm, mean ± SD	0.7 ± 0.1	0.7 ± 0.2	0.11	0.8 ± 0.2	0.7 ± 0.1	0.11	0.7 ± 0.1	0.7 ± 0.1	0.45

SD: standard deviation; ET: erythematotelangiectatic subtype; PP: papulopustular subtype; BMI: body mass index; CRP: C-reactive protein; NLR: neutrophil-to-lymphocyte ratio; PLR: platelet-to-lymphocyte ratio; MPV: mean platelet volume; VAT: visceral adipose tissue.

**Table 3:** Multivariable linear regression analyses of significant parameters.

Independent predictors for *preperitoneal fat* by multivariable linear regression analysis; Rosacea vs. Control					
Variables	Estimate	Standard Error	%95 CI	t	*p* Value
Age, year	0.06	0.03	0.01–0.11	1.99	0.04
Gender	0.03	0.8	−1.56–1.62	0.03	0.97
BMI, kg/m^2^	0.67	0.11	0.44–0.89	5.85	0.01
Presence of Rosacea	3.22	0.52	2.18–4.27	6.12	0.01
Independent predictors for *aorta VAT* by multivariable linear regression analysis; Rosacea vs. Control					
Variables	Estimate	Standard Error	%95 CI	t	*p* Value
Age, year	0.21	0.09	0.04–0.39	2.45	0.01
Gender	5.21	2.58	0.11–10.33	2.02	0.04
BMI, kg/m^2^	1.97	0.37	1.24–2.70	5.36	0.01
Presence of Rosacea	11.67	1.69	8.33–15.02	6.91	0.01
Independent predictors for *preperitoneal fat* by multivariable linear regression analysis, in the group of rosacea patients					
Variables	Estimate	Standard Error	%95 CI	t	*p* Value
Age, year	0.04	0.04	−0.04–0.13	0.95	0.34
Gender	0.85	1.29	−1.73–3.43	0.65	0.51
BMI, kg/m^2^	0.79	0.19	0.42–1.17	4.22	0.01
Rosacea Severity	1.84	1	−0.16–3.85	1.83	0.07
Independent predictors for perirenal *VAT* by multivariable linear regression analysis, in the group of rosacea patients					
Variables	Estimate	Standard Error	%95 CI	t	*p* Value
Age, year	0.05	0.02	0.01–0.08	2.96	0.01
Gender	0.28	0.49	−0.69–1.25	0.58	0.56
BMI, kg/m^2^	0.33	0.07	0.19–0.47	4.66	0.01
Rosacea Severity	0.26	0.38	−0.49–1.01	0.69	0.49

CI: confidence interval; BMI: body mass index; VAT: visceral adipose tissue.

**Table 4:** Summary of the key findings.

		Rosacea Group (n = 73)		Control Group (n = 73)		*p* Value
ESR mm/first h, mean ± SD		15.10 ± 10.8		6.86 ± 3.05		<0.01
Preperitoneal fat—xiphoid, mm, mean ± SD		14.11 ± 4.47		11.0 ± 3.12		<0.01
Aorta VAT, mm, mean ± SD		36.9 ± 16.26		25.57 ± 6.32		<0.01
		**Mild Rosacea (n = 19)**		**Moderate–Severe Rosacea (n = 54)**		***p* Value**
Age, year, mean ± SD		32.8 ± 9.4		39.4 ± 11.5		0.02
BMI, kg/m^2^, mean ± SD		23.8 ± 2.8		25.3 ± 2.5		0.03
Preperitoneal fat—xiphoid, mm, mean ± SD		11.6 ± 4.2		14.9 ± 4.2		<0.01
Perirenal VAT, mm, mean ± SD		7.4 ± 1.4		8.5 ± 1.9		0.049
**Multivariable Linear Regression Analysis**						
		**Estimate**	**Standard Error**	**%95 CI**	**t**	***p* Value**
Predictors for **preperitoneal fat**, Rosacea vs. Control	Age, year	0.06	0.03	0.01–0.11	1.99	0.04
	BMI, kg/m^2^	0.67	0.11	0.44–0.89	5.85	0.01
	Presence of Rosacea	3.22	0.52	2.18–4.27	6.12	0.01
Predictors for aorta **VAT**, Rosacea vs. Control	Age, year	0.21	0.09	0.04–0.39	2.45	0.01
	Gender	5.21	2.58	0.11–10.33	2.02	0.04
	BMI, kg/m^2^	1.97	0.37	1.24–2.70	5.36	0.01
	Presence of Rosacea	11.67	1.69	8.33–15.02	6.91	0.01

ESR: erythrocyte sedimentation rate; SD: standard deviation; VAT: visceral adipose tissue; BMI: body mass index; CI: confidence interval.

## Data Availability

The data featured in this work can be obtained upon request to the corresponding author.
